# An Increase in Dietary Net Energy Concentration Affects Nutrient Digestibility and Noxious Gas Emissions and Reveals a Better Growth Rate in Growing–Finishing Pigs

**DOI:** 10.3390/ani15182761

**Published:** 2025-09-22

**Authors:** Usman Kayode Kolawole, In Ho Kim

**Affiliations:** 1 Department of Animal Biotechnology, Dankook University, Cheonan 31116, Republic of Korea; kolawoleusman@rocketmail.com; 2 Smart Animal Bio Institute, Dankook University, Cheonan 31116, Republic of Korea

**Keywords:** nutrient utilization, energy variation, growing–finishing pig, growth performance

## Abstract

This experiment was performed to evaluate the optimum level of net energy (NE) to improve growth performance and nutrient digestibility, and reduce noxious gas emissions in growing–finishing pigs. A total of 150 pigs ([Yorkshire × Landrace] × Duroc) with an initial average body weight (BW) of 32.64 ± 1.49 kg were randomly assigned to one of five treatments for 112 days (16 weeks: growing stage, initial–week 6; finishing stage, 6-finish/week 16). There were five treatment groups with six replicates and five pigs (three males and two females) per pen. The dietary treatment includes CON, a basal diet (NE 2475 kcal/kg), TRT1, basal diet −5.0% NE (2353 kcal/kg), TRT2, basal diet −2.5% NE (2414 kcal/kg), TRT3, basal diet +2.5% NE (2537 kcal/kg), and TRT4, basal diet +5.0% NE (2599 kcal/kg). Through the experiment, increasing NE by +5.0% increased average daily gain and bodyweight, having the highest value (*p* < 0.05) and a decreased feed conversion ratio (*p* < 0.05), whereas decreasing NE by −5% decreased average daily gain (*p* < 0.05) and increased feed conversion ratio (*p* < 0.05) at week 6 with no effects on nutrient digestibility and noxious gas emission. Hence, integrating the increasing level of net energy (NE) with +5.0% into the pig diet during the growing–finishing phase can be considered the appropriate approach for enhancing both average daily gain and feed efficiency in pigs. A +5% (2599 kcal/kg) increase in NE has the potential to reduce costs by lowering the amount of feed needed while either sustaining or improving growth, as the use of energy-dense ingredients becomes more cost effective.

## Simple Summary

Pig producers can adjust feeding practices and compositions of diets to maximize profitability in the swine production sector by increasing their knowledge of the link between accessibility of nutrients and efficiency in production. The energy that is actually accessible to the animals for production and management is represented by the net energy. When evaluating a pig’s energy intake and its effect on performance, net energy is thought to be the most accurate method. In order to make a discernible difference in growth performance and nutrient digestibility, a wider variety of dietary energy levels might be required. The key to pigperformance may be dietary changes to guarantee adequate net energy content. This study investigated the optimum level of net energy (NE) to im-prove growth performance and nutrient digestibility, and reducenoxious gas emissions in growing–finishing pigs. The study results confirmed that increasing the level of NE by 5.0% in the diet of growing–finishing pigs can be favorable to producers for the average daily gain and feed efficiency. Raising net energy (NE) in swine feed can lower feeding expenses by reducing the total feed quantity necessary for each unit of weight gain and enhancing feed conversion efficiency, resulting in greater profitability and a rise in income relative to feed costs.

## 1. Introduction

The costs of ingredients providing energy for pigs contribute the largest portion of total feed costs [1,2], and it has been evaluated that costs of feed make up 70% of total costs for livestock production [3] and the energy component constitutes the greatest proportion. Accurate evaluation of the energy value of feeds and adjusting the diet formulations is vital, both for least-cost formulation goals and adjusting feed supply to the energy requirements of animals [1,2]. Apart from increasing profit, the outcome of this formulation on performance, nutrient intake, digestibility, and environmental footprints is vital [4].

The energy system, which is known to have three systems with a balanced value of feed energy, includes NE. The typical denominator of energy usage in pigs is followed by every energy system, as described by NRC [5]. Although the NE system has been studied for decades, recent applied studies on its effects in swine production are limited [6,7]. The NE system, which is recognized as the approximate determinant of components and values of dietary energy for the reason that it takes the heat increment from the digestive process and metabolism of feeds into account, gives better suggestions on pig performance because it shows the available energy to pigs [8,9]. A greater NE content in the diet can improve the effectiveness of energy use for growth [10]. More fiber normally decreases overall nutrient digestibility, and a higher NE diet may have less fiber, which can improve the digestion of other nutrients [11]. High energy levels have been known to increase the digestibility of ether extract in diets [11]. Emissions are reduced by diets with higher net energy and frequently increased by diets with lower net energy [12]. However, the acceptance, understanding, and usage of the NE system are low. NE is hard to determine and more complex than other systems. Moreover, there are factors the NE system has been known to be impacted by, which include variation among pigs and growth stage [13]. Therefore, factors that influence the energy requirement for maintenance and the capability of pigs to digest and utilize nutrients should be considered.

Most of the available research on net energy (NE) is old, and recent research is rare withnot as many as the old ones, and a little change in energy might not increase the cost. There have been limitations in observing the effects of dietary energy density on the performance of pigs [14]. Some previous research on the net energy system has focused on understanding NE, equation prediction [15], usage, usefulness, and slow adoption [14], other than the feeding estimation of dietary NE, which has increased and there are decreased levels in growing to finishing pigs for reduction in feed cost.

We hypothesized that altering NE levels in pig diets would influence growth performance, nutrient digestibility, and noxious gas emissions. Therefore, this study aimed to observe varying levels of NE to find the optimum level of NE to improve growth performance, nutrient digestibility, and noxious gas emissions in growing–finishing pigs.

## 2. Materials and Methods

All the procedures used in the experiment were revised and accepted by the Institutional Animal Care and Use Committee of Dankook University, Republic of Korea (Ethical approval No. DK-2-2307).

### 2.1. Animal Husbandry, Diet, Experimental Design

In a 112-day trial, a total of 150 crossbred growing pigs ([Yorkshire × Landrace] × Duroc) were used in this experiment with an initial BW of (32.65 ± 1.49) kg and assigned. The pigs were randomly assigned to one of five nutritional treatments according to their initial BW. Each treatment comprised of six replicates with mixed-sex of 2 gilts and 3 barrows per pen. The dietary treatment included CON, a basal diet (NE 2475 kcal/kg), TRT1, basal diet −5.0% NE (2353 kcal/kg), TRT2, basal diet −2.5% NE (2414 kcal/kg), TRT3, basal diet +2.5% NE (2537 kcal/kg), and TRT4, basal diet +5.0% NE (2599). There were two stages of the experiment: the growing stage (initial–6 weeks) and the finishing stage (weeks 6–finish/16, which is divided into 2 phases: phase 1, weeks 6–12; and phase 2, weeks 12–16). Pigs were kept in an environmentally controlled room equipped with a mechanical aeration system. In the barn, slatted plastic base was used for the floor construction. Throughout the experiment, pigs had *ad libitum* access to water and feed from pens equipped with a nipple drinker and feeder. The basal diets formulation in two (2) stages in the experiment (Table 1, Table 2 and Table 3) was carried out according to the 2475 kcal/kg in the guidelines of NRC [5], while treatment diets had variationsin NE than the recommendation of NRC [5]. The other nutrients were maintained to meet or exceed the NRC [5] estimates of requirements.

### 2.2. Sampling Collections

#### 2.2.1. Growth Performance

The individual body weight of pigs was measured on initial, 6, 12 and 16 weeks to calculate the average daily gain (ADG) on pen-based and feed intake was checked daily to calculate the average daily feed intake (ADFI). The feed conversion ratio (FCR) was calculated using ADG and ADFI values.

#### 2.2.2. Nutrient Digestibility

All pigs were given a diet containing 0.3% chromium oxide (Cr_2_O_3_), an indigestible marker to ascertain the nutrient digestibility of dry matter (DM), nitrogen (N), and energy (E), seven days before fecal sample collection (i.e., from the end of week 5 to 6). Fresh fecal samples were obtained by rectal palpation from a minimum of two pigs each pen (one male and one female) at the end of week six. They were then homogenized and taken to the Smart Animal Bio Institute laboratory, Dankook University where they were kept at −20 °C until additional analysis. Fecal samples were kept for three days at 105 °C in a convection oven before examination. The samples were then crushed and run through a sieve measuring 1.2 mm. According to Association of Official Analytical Chemists (AOAC) [16], the nutritional digestibility of N (method 984.13A-D, AOAC, 2006) and DM (method 934.01, AOAC, 2006) in feed and fecal samples was investigated [16]. UV absorption spectrophotometry was used to investigate chromium (Shimadzu, UV-1201, Shimadzu, Kyoto, Japan). A bomb calorimeter (Parr 6100; Parr Instrument Co., Moline, IL, USA) was used to measure the samples’ heat of combustion in order to calculate the E. A Kjeltec 2300TM Analyzer (Foss Tecator AB, Hoeganaes, Sweden) was used to ascertain N [17].

Formula for ATTD:

Nutrient digestibility (%) = [1 − (Nf × Cd)/(Nd × Cf)] × 100, where Nf—number of nutrients in feces (% DM), Nd number of nutrients in the diet (% DM), Cd—chromium content in the diet (% DM), and Cf—chromium content in feces (% DM) [18].

#### 2.2.3. Noxious Gas Emissions

On day 42, two pigs were randomly selected from each pen to collect freshly voided fecal samples (300 g). The fecal samples were placed in a 2.6 L plastic box with small holes on one side and sealed with tape. For 7 days, the boxes were kept at room temperature for the fermentation process to occur. After fermentation, the gas detection was performed using a MultiRAE Lite PGM-6208 (RAE, Sunnyvale, CA, USA) to measure the levels of ammonia (NH_3_), hydrogen sulfide (H_2_S), methyl mercaptans, acetic acid and carbon dioxide (CO_2_). To measure, a hole was made in the adhesive tape covering the box, and 100 mL of air from approximately two centimeters above the fecal surface was sampled. Each box was then resealed with adhesive tape after air sampling. After 48 h, the measurements were performedagain, and the gas contents were decided by averaging two readings from the same box [19].

### 2.3. Statistical Analysis

The SAS (2008) statistical software package’s General Linear Model technique was used to analyze all of the data. Net energy, gender, and the combination of net energy and gender were fixed effects in the statistical model, while pen was a random effect. The statistical difference of treatment, gender, and interaction effects with *p* ≤ 0.05 were deemed significant. When the *p*-Value was 0.05 or less, the Tukey test was used to compare the means.

## 3. Results

### 3.1. Growth Performance

The effect of varying levels of net energy on growth performance was presented in Table 4. Through the experimental period, pigs were fed diets with varying levels of low and high NE of−2.5%, −5.0%, +2.5% and +5.0%. At week 6, significant differences were observed in the bodyweight; as the NE increases by 2.0% and 5.0% the bodyweight increases, and as the NE decreases by 2.0% and 5% the bodyweight decreases compared to the control group. At week 6, significant differences were observed in the ADG and FCR (*p* = 0.017 and *p* = 0.002), increasing the NE value by 2.0% and 5.0% increases the ADG and decreases the FCR, but the highest ADG and lowest FCR were recorded when NE is increased by 5.0%. Furthermore, reducing NE by 2.0% and 5.0% decreases the ADG and increases the FCR. There was no significant difference was observed on the ADFI and after week 6 on ADG, FCR, and ADFI.

### 3.2. Nutrient Digestibility

There was no significant difference in the apparent total tract digestibility (ATTD) of dry matter (DM), nitrogen (N) and apparent retention of energy (E), shown on Table 5.

### 3.3. NoxiousGas Emissions

There was no significant difference in ammonia (NH_3_), hydrogen sulfide (H_2_S), methyl mercaptans, acetic acid and carbon dioxide (CO_2_), shown on Table 6.

## 4. Discussion

There are various ways to express energy demand; they primarily involve adjusting the diet energy density for pigs fed ad libitum based on criteria such as pig development potential, feed intake regulation, or financial concerns [6,7]. At the old stage of pigs, they are known to eat to fulfill the required energy. According to Zijlstra and Beltranena [20], energy losses from heat generation and variations in nutrition metabolic use are not taken into consideration by other energy systems of digestible energy and metabolizable energy systems; a more precise estimate of the pig’s energy availability is provided by the NE system. It has been reported that pig performance are influence in various ways at different phases of growth by changes in energy density [5]. In this experiment, the dietary NE had effects on growth performance in growing–finishing pigs at the week 6 of the trial. Pigs fed diets with an increase in dietary NE by 5.0% had an increase in ADG and BWG but decrease in FCR, while the pigs fed with reduction in dietary NE by 5.0% had decrease in ADG and increase in FCR. As the dietary NE increase or decrease the ADG and FCR change in response to the varying NE levels. Our findings are consistent with previous reports [21,22] showing that higher dietary NE improves body weight gain. Pigs fed diets with higher dietary NE have increased ADG and elevation in efficiency of feed [15,23], while the feed intake is reduced [24,25,26]. Increasing of NE from 2352 to 2599 kcal/kg NE (increased NE by +5.0%) significantly influenced the ADG and FCR by increasing ADG and decreasing the FCR. Without substantially altering feed intake, a greater NE level in the diet can improve the effectiveness of energy usage for growth [2]. There have been reports in finishing pigs of increase dietary NE not having effect on ADFI [27] which is noticeable among the treatments; the overall FI showed no observable or significant influence by the dietary NE [28]. Therefore, increasing dietary NE may be useful for improving pig growth during the growing–finishing phase. Enhancing net energy (NE) in pig feed can lower feed expenses by reducing the overall quantity of feed required for each unit of weight gain and by improving feed conversion efficiency. This results in greater profitability and an increase in income relative to feed costs.

In addition to managing energy availability, feed makers can indirectly impact nutritional digestibility by regulating dietary NE levels through ingredient selection [29]. Nutrient digestibility and NE can have a complicated relationship that is frequently impacted by interactions between various feed ingredients [30]. While more fiber normally decreases overall nutrient digestibility, a higher NE diet may have less fiber, which can improve the digestibility of other nutrients [29]. The experiment shows that there was no observable difference in nutrient digestibility between groups. Dietary energy content and digestibility of nutrients are impacted by the composition of diets as the significant determinants [31]. Park [11] reported that 2.32 to 2.54 kcal/kg NE in diets of pigs has increased ATTD of acid-hydrolyzed ether extraction (AEE); and Lee [24] showed that increasing NE concentrations in diets from 8.0 to 12.0 MJ/kg increased ATTD of DM. Crude protein (CP) and organic matter were increased, ATTD of nutrients increased with increasing dietary NE level. In contrast, Liu [12] found no effect in energy deficiency on nutrient digestibility of growing pigs. The reduction in energy in those studies [12] was 2 to 5% of basal diet. Therefore, the effect of net energy on nutrient digestibility depends on the higher deficiency of NE and a 2.5 to 5% variation in NE could not affect nutrient digestibility in pigs.

In order to reach the desired net energy value, higher energy diets frequently call for lower levels of protein and carbohydrates. This lowers the amount of substrates (such as fermentable carbohydrates and undigested proteins) available for microbial fermentation in the hindgut, which eventually results in a decrease in the release of gases like volatile fatty acids and ammonia. Emissions decrease with greater net energy diets and increase with lower net energy diets [12]. This is due to the fact that diets with a higher net energy content facilitate better digestion and utilization of nutrients, which means that less undigested material enters the stomach to be fermented by bacteria and produce gasses such as hydrogen sulfide (H_2_S), ammonia (NH_3_), and methane [12]. The experiment shows that there was no difference of noxious gas emissions in between groups. This is in agreement with other studies on this, that there is no difference in control diets and energy level reduction diets on some noxious gas emission like NH_3_ and H_2_S in growing pigs [32]; low and high dietary energy have no effect on noxious gas emissions [33]. Nitrogen indigestibility is the major source of NH_3_ in finishing pigs [34]. Gas emissions in monogastric animals depend on nutrient digestibility and microbial fermentation [35,36]. As our study showed no changes in nutrient digestibility, no effect on gas emissions is reasonable. Dissimilarity with other reports may be due to the level of NE, fiber content, composition and management of diets and pig breeds.

## 5. Conclusions

In conclusion, varying levels of NE at −5.0% (2353 kcal/kg), −2.5% (2414 kcal/kg), +2.5% NE (2537 kcal/kg), +5.0% NE (2599 kcal/kg) on growth performance, nutrient digestibility and noxious gas emissions have an effect on growth performance of growing–finishing pigs, increasing NE, specifically by 5% (2599 kcal/kg) in the diets, showing a beneficial effect on ADG and BWG of growing–finishing pigs, while decreasing by 5.0% NE (2352 kcal/kg) decreased ADG and BWG. However, varying levels of NE have no significant effect on digestibility of DM, N, and E, and emissions of ammonia (NH_3_), hydrogen sulfide (H_2_S), methyl mercaptans, acetic acid and carbon dioxide (CO_2_). A +5% (2599 kcal/kg) rise in net energy might cut costs by using less feed while keeping growth the same or even better, as energy-rich ingredients become more economically viable. This may be beneficial in diet formulation regarding pig production. Further studies may be needed on this.

## Figures and Tables

**Table 1:** Composition of Growing Pig Diets ^1^.

Item	Experimental Diet				
	CON	NE −5.0%	NE −2.5%	NE +2.5%	NE +5.0%
Ingredients (%)					
Corn	76.44	64.71	70.56	75.71	73.88
Soybean meal	19.16	16.83	18.00	19.60	19.93
Palm kernel meal	1.00	15.00	8.00	-	-
Tallow	0.05	0.16	0.12	1.35	2.85
MDCP	1.48	1.30	1.40	1.48	1.48
Limestone	0.76	0.82	0.78	0.76	0.77
Salt	0.20	0.20	0.20	0.20	0.20
Methionine (99%)	0.05	0.06	0.05	0.05	0.05
Lysine (78%)	0.49	0.49	0.46	0.42	0.41
Mineral mix ^2^	0.20	0.20	0.20	0.20	0.20
Vitamin mix ^3^	0.20	0.20	0.20	0.20	0.20
Choline (25%)	0.03	0.03	0.03	0.03	0.03
Total	100.00	100.00	100.00	100.00	100.00
Calculated value					
Crude protein, %	16.00	16.00	16.00	16.00	16.00
NE (kcal/kg) ^4^	2352	2414	2475	2537	2599
ME (kcal/kg) ^5^	3216	3248	3300	3341	3407
Calcium, %	0.70	0.70	0.70	0.70	0.70
Phosphorus, %	0.60	0.60	0.60	0.60	0.60
Lysine, %	1.10	1.10	1.10	1.10	1.10
Methionine, %	0.30	0.30	0.30	0.30	0.30
Fat, %	2.66	2.84	2.99	4.25	5.67

^1^ A grow-to-finishing feeding procedure designed to either meet or exceed the NRC’s [5] suggested standards. ^2^ Provided per kg diet: Fe, 100 mg as ferrous sulfate; Cu, 17 mg as copper sulfate; Mn, 17 mg as manganese oxide; Zn, 100 mg as zinc oxide; I, 0.5 mg as potassium iodide; and Se, 0.3 mg as sodium selenite. ^3^ Provided per kilograms of diet: vitamin A, 10,800 IU; vitamin D3, 4000 IU; vitamin E, 40 IU; vitamin K3, 4 mg; vitamin B1, 6 mg; vitamin B2, 12 mg; vitamin B6, 6 mg; vitamin B12, 0.05 mg; biotin, 0.2 mg; folic acid, 2 mg; niacin, 50 mg; D-calcium pantothenate, 25 mg; ME, Metabolizable Energy; NE, net energy; MDCP, monodicalcium phosphate. ^4^ Net energy determined through equations 1 to 8 from the NRC [5]. ^5^ Metabolizable energy determined through equations 1 to 5 from the NRC [5].

**Table 2:** Composition of Finishing Pig Diets (Phase 1) ^1^.

Item	Experimental Diet				
	CON	NE −5.0%	NE −2.5%	NE +2.5%	NE +5.0%
Ingredients (%)					
Corn	76.77	65.02	70.89	79.33	77.45
Soybean meal	16.15	13.83	14.98	16.95	17.28
Palm kernel meal	4.00	18.00	11.00	-	-
Tallow	0.13	0.24	0.20	0.75	2.28
MDCP	1.20	1.05	1.10	1.24	1.28
Limestone	0.66	0.70	0.70	0.66	0.64
Salt	0.20	0.20	0.20	0.20	0.20
Methionine (99%)	0.06	0.07	0.07	0.06	0.06
Lysine (78%)	0.40	0.46	0.43	0.38	0.38
Mineral mix ^2^	0.20	0.20	0.20	0.20	0.20
Vitamin mix ^3^	0.20	0.20	0.20	0.20	0.20
Choline (25%)	0.03	0.03	0.03	0.03	0.03
Total	100.00	100.00	100.00	100.00	100.00
Calculated value					
Crude protein, %	15.00	15.00	15.00	15.00	15.00
NE (kcal/kg) ^4^	2352	2414	2475	2537	2599
ME (kcal/kg) ^5^	3300	3251	3282	3328	3395
Calcium, %	0.60	0.60	0.60	0.60	0.60
Phosphorus, %	0.55	0.55	0.55	0.55	0.55
Lysine, %	1.00	1.00	1.00	1.00	1.00
Methionine, %	0.30	0.30	0.30	0.30	0.30
Fat, %	2.70	2.88	3.04	3.75	5.19

^1^ A grow-to-finishing feeding procedure designed to either meet or exceed the NRC’s [5] suggested standards. ^2^ Provided per kg diet: Fe, 100 mg as ferrous sulfate; Cu, 17 mg as copper sulfate; Mn, 17 mg as manganese oxide; Zn, 100 mg as zinc oxide; I, 0.5 mg as potassium iodide; and Se, 0.3 mg as sodium selenite. ^3^ Provided per kilograms of diet: vitamin A, 10,800 IU; vitamin D3, 4000 IU; vitamin E, 40 IU; vitamin K3, 4 mg; vitamin B1, 6 mg; vitamin B2, 12 mg; vitamin B6, 6 mg; vitamin B12, 0.05 mg; biotin, 0.2 mg; folic acid, 2 mg; niacin, 50 mg; D-calcium pantothenate, 25 mg; ME, Metabolizable Energy; NE, net energy; MDCP, monodicalcium phosphate. ^4^ Net energy determined through equations 1 to 8 from the NRC [5]. ^5^ Metabolizable energy determined through equations 1 to 5 from the NRC [5].

**Table 3:** Composition of Finishing Pig Diets (Phase 2) ^1^.

Item	Experimental Diet				
	CON	NE −5.0%	NE −2.5%	NE +2.5%	NE +5.0%
Ingredients (%)					
Corn	72.71	68.10	78.82	84.52	83.83
Soybean meal	10.26	8.04	9.10	11.43	11.86
Palm kernel meal	8.00	21.00	15.00	1.00	-
Tallow	0.18	0.05	0.24	0.15	1.42
MDCP	1.00	0.85	0.95	1.10	1.10
Limestone	0.66	0.70	0.66	0.64	0.64
Salt	0.20	0.20	0.20	0.20	0.20
Methionine (99%)	0.09	0.10	0.10	0.09	0.09
Lysine (78%)	0.47	0.53	0.50	0.44	0.43
Mineral mix ^2^	0.20	0.20	0.20	0.20	0.20
Vitamin mix ^3^	0.20	0.20	0.20	0.20	0.20
Choline (25%)	0.03	0.03	0.03	0.03	0.03
Total	100.00	100.00	100.00	100.00	100.00
Calculated value					
Crude protein, %	13.00	13.00	13.00	13.00	13.00
NE (kcal/kg) ^4^	2352	2414	2475	2537	2599
ME (kcal/kg) ^5^	3208	3251	3276	3307	3369
Calcium, %	0.55	0.55	0.55	0.55	0.55
Phosphorus, %	0.50	0.50	0.50	0.50	0.50
Lysine, %	0.90	0.90	0.90	0.90	0.90
Methionine, %	0.30	0.30	0.30	0.30	0.30
Fat, %	2.53	2.90	3.06	3.25	4.49

^1^ A grow-to-finishing feeding procedure designed to either meet or exceed the NRC’s [5] suggested standards. ^2^ Provided per kg diet: Fe, 100 mg as ferrous sulfate; Cu, 17 mg as copper sulfate; Mn, 17 mg as manganese oxide; Zn, 100 mg as zinc oxide; I, 0.5 mg as potassium iodide; and Se, 0.3 mg as sodium selenite. ^3^ Provided per kilograms of diet: vitamin A, 10,800 IU; vitamin D3, 4000 IU; vitamin E, 40 IU; vitamin K3, 4 mg; vitamin B1, 6 mg; vitamin B2, 12 mg; vitamin B6, 6 mg; vitamin B12, 0.05 mg; biotin, 0.2 mg; folic acid, 2 mg; niacin, 50 mg; D-calcium pantothenate, 25 mg; ME, Metabolizable Energy; NE, net energy; MDCP, monodicalcium phosphate. ^4^ Net energy determined through equations 1 to 8 from the NRC [5]. ^5^ Metabolizable energy determined through equations 1 to 5 from the NRC [5].

**Table 4:** The Effect of Dietary Feed on Growth Performance in Growing–Finishing Pigs ^1^.

Items	CON	TRT1	TRT2	TRT3	TRT4	Gender		SEM ^2^	*p*-Value		
						Barrow	Gilt		NE	Gender	Interaction
Body weight, kg											
Buffering	24.67	24.67	24.67	24.67	24.66	24.67	24.67	0.06	0.001	0.128	0.246
Initial	32.65	32.64	32.64	32.65	32.65	32.65	32.64	0.07	0.101	0.245	0.386
Week 6	51.34 ^ab^	50.14 ^b^	50.62 ^ab^	51.64 ^a^	51.78 ^a^	51.64	51.63	3.44	0.018	0.061	0.075
Finish	111.78	111.46	112.67	113.12	114.64	111.72	111.69	8.433	0.189	0.873	0.959
Buffering–Initial											
ADG, g	511	509	508	515	506	508	506	12	0.635	0.719	0.778
ADFI, g	1063	1059	1055	1060	1053	1060	1059	20	0.803	0.939	0.642
FCR	2.080	2.085	2.077	2.062	2.082	2.083	2.084	0.017	0.331	0.492	0.577
Initial–Week 6											
ADG, g	697 ^ab^	655 ^b^	673 ^ab^	706 ^ab^	715 ^a^	706	702	17	0.017	0.078	0.265
ADFI, g	1657	1595	1620	1643	1654	1644	1643	25	0.094	0.205	0.108
FCR	2.380 ^ab^	2.438 ^a^	2.411 ^a^	2.329 ^b^	2.314 ^b^	2.398	2.402	0.026	0.002	0.064	0.076
Week 6–Finish											
ADG, g	863	876	886	878	898	897	894	22	0.236	0.540	0.585
ADFI, g	2561	2582	2603	2599	2633	2614	2614	39	0.188	0.285	0.323
FCR	2.970	2.951	2.940	2.961	2.935	2.948	2.952	0.032	0.352	0.073	0.094
Overall											
ADG, g	778	775	786	790	803	788	786	17	0.218	0.345	0.586
ADFI, g	1970	1974	1990	1991	2013	1989	1982	26	0.225	0.268	0.408
FCR	2.535	2.550	2.535	2.523	2.508	2.528	2.532	0.022	0.169	0.243	0.118

^1^ Abbreviation: CON, a basal diet (NE 2475 kcal/kg), TRT1, basal diet −5.0% NE (2353 kcal/kg), TRT2, basal diet −2.5% NE (2414 kcal/kg), TRT3, basal diet +2.5% NE (2537 kcal/kg), and TRT4, basal diet +5.0% NE (2599 kcal/kg). ^2^ Standard error of means. ^a,b^ Means in the same row with different superscripts differ (*p* < 0.05).

**Table 5:** The Effect of Dietary Feed on Nutrient Digestibility in Growing–Finishing Pigs ^1^.

Items	CON	TRT1	TRT2	TRT3	TRT4	Gender		SEM ^2^	*p*-Value		
						Barrow	Gilt		NE	Gender	Interaction
**Week 6**											
Dry matter	76.89	76.42	76.55	76.95	77.23	76.88	76.85	0.47	0.244	0.571	0.236
Nitrogen	73.96	73.94	74.02	74.30	74.24	73.94	73.94	0.28	0.381	0.212	0.765
Energy	75.49	75.02	75.33	75.63	75.80	75.06	75.02	0.53	0.314	0.534	0.568

^1^ Abbreviation: CON, a basal diet (NE 2475 kcal/kg), TRT1, basal diet −5.0% NE (2353 kcal/kg), TRT2, basal diet −2.5% NE (2414 kcal/kg), TRT3, basal diet +2.5% NE (2537 kcal/kg), and TRT4, basal diet +5.0% NE (2599 kcal/kg). ^2^ Standard error of means.

**Table 6:** The Effect of Dietary Feed on Gas Emission in Growing–Finishing Pigs ^1^.

Items	CON	TRT1	TRT2	TRT3	TRT4	Gender		SEM ^2^	*p*-Value		
						Barrow	Gilt		NE	Gender	Interaction
**Week 6**											
NH_3_	6.13	6.13	6.63	5.88	5.75	6.18	6.15	0.30	0.058	0.602	0.184
H_2_S	5.03	5.73	5.13	4.65	4.98	5.73	5.74	0.44	0.109	0.078	0.206
Methyl mercaptans	5.38	6.38	5.50	5.50	6.00	5.50	5.47	0.78	0.382	0.548	0.108
Acetic acid	10.13	11.38	10.63	10.75	10.75	10.12	10.10	1.41	0.542	0.782	0.904
CO_2_	13,275	13,375	12,925	12,825	12,900	12,889	12,886	383	0.330	0.620	0.882

^1^ Abbreviation: CON, a basal diet (NE 2475 kcal/kg), TRT1, basal diet −5.0% NE (2353 kcal/kg), TRT2, basal diet −2.5% NE (2414 kcal/kg), TRT3, basal diet +2.5% NE (2537 kcal/kg), and TRT4, basal diet +5.0% NE (2599 kcal/kg). ^2^ Standard error of means.

## Data Availability

Data are available on request due to privacy.
